# Defects detection of GMAW process based on convolutional neural network algorithm

**DOI:** 10.1038/s41598-023-48698-x

**Published:** 2023-12-01

**Authors:** Haichao Li, Yixuan Ma, Mingrui Duan, Xin Wang, Tong Che

**Affiliations:** State Key Laboratory of Advanced Welding and Joining, Harbin Institute of Technology, Harbin, 150001 China

**Keywords:** Design, synthesis and processing, Computer science

## Abstract

It is significant to predict welding quality during gas metal arc welding process. The welding defect detection algorithm has been developed based on convolutional neural network (CNN). The sensing system and image processing algorithm for molten pools has been developed. It overcomes the interference caused by the arc light to obtain clear images of the molten pool's boundaries. The molten pools images are used to build up training set and test set for training and testing the CNN model. The model is designed to extract the visual features of molten pool images to predict the penetration state, the welding crater, and slags. Through optimizing the network parameters such as kernel-size, batch-size and learning rate, the prediction accuracy is higher than 95%. Moreover, the model enhances additional focus on the welding crater based on the welder experience. The mechanisms between molten pool characteristics and welding defects were analyzed based on the welder experience and the visual features of the model. It is found that the model judges the occurrence of burn-through with the black hole in the middle zone of the molten pool. When the surface pores are generated, the model exhibits a strong response to circular voids in the semi-solid region at the trailing end of the molten pool. The size and shape of fusion holes exhibit a strong correlation with the molten state. When the shape of the crater does not appear concave, it often signifies excessive penetration. It contributes to enhancing the algorithm's robustness during various welding scenarios.

## Introduction

Intelligent robotic Welding Manufacturing (IWM) has been extensively applied in the field of energy equipment, shipbuilding, nuclear power construction and aerospace. Gas metal arc welding (GMAW)is one of the most important welding technologies due to the efficiency and high-quality. In GMAW process, there are some common weld defects such as burn through, lack of fusion, slag and surface pores. The real-time defect detection contributes to improving welding quality for robotic welding process^1^. The traditional welding quality inspection method is to detect the weld defects after welding through ultrasonic, X-ray testing and other non-destructive testing methods. These method is widely applied for inspecting welding quality. However, these post-weld inspection methods lack the capacity to proactively address welding defects. The real-time defeat detection of welding quality provides real-time feedback on defects during the welding process, contributing to the enhancement of welding quality.

The welding process and welding defects are monitored based on various sensing technologies such as current sensing^2^, voltage sensing^3^, acoustic sensing^4^,and visual sensing^5–7^. However, the current sensing and voltage sensing are unable to sense molten pool information. The acoustic sensing is susceptible to the environment noise during welding process. The visual sensor is able to obtain comprehensive molten pool information and exhibit a high sensing accuracy. The welding process information with visual sensing is obtained widely, providing the data for achieving closed-loop control of the welding process^8^.H Cao et al.^9^ used infrared visual sensor to detect defects on TIG welding. However, the molten pool defects are unable to be detected accurately due to the molten pool temperature falling outside the range of infrared imaging temperature. Gao Jinqiang et al.^10^ used the coaxial vision sensing to detect the molten pool and keyhole in the laser welding process. However, the coaxial sensors is not applicable to GMAW due to differences in the welding torch structure and arc morphology. It’s a challenge to obtain comprehensive molten pool information for GMAW. Besides, most visual sensing systems rely on low dimensional image features to predict welding quality. It’s difficult to extract high dimensional features.

Deep learning algorithms are employed to extract high-dimensional visual features of the molten pool. Ario sunar baskoro et al.^11^ developing the artificial neural network (ANN) to extract molten pool geometric features for welding quality control. However, this method faces challenges in achieving high-precision detection when dealing with various types of welding defects. Kovacevic et al^12^ established the model of weld pool size information and back weld width based on classic neural network. This approach has limited accuracy in detecting welding defects due to the algorithm's capacity for dimensionality feature extraction. Liu Xinfeng et al.^13^ utilized the extracted features such as length, width, area and trailing angle of the weld pool as the input of the neural network. The proposed neural network model accurately predict the change of weld pool back weld width. However, the method is unable to detect welding defects accurately. Convolution neural network (CNN) is a kind of feedforward neural network with deep structure, which gives better results in image classification and target recognition^14^. He Deqiang^[15]^proposed an intelligent model for welding quality detection based on attention balanced context Mask R-CNN. The model predicts welding defects accurately. But it is unfeasible to perform real-time detection due to relying on ultrasonic inspection images. Zhang ZF^16^ studied deep learning-based on-line defects detection for aluminum alloy in robotic arc welding using CNN and weld images. The model predicts welding defects accurately. However, it lacks an analysis of the welding mechanisms of defect prediction. Guo B^[17]^proposed a welding defect classification method based on lightweight CNN. The feature data of each convolution layer are visualized to verify the feasibility of the model and improve the interpretability of the model. However, it lacks robustness across various welding scenarios. These models^18–20^ achieve high accuracy in predicting defects within a specific scenario based on CNN and its improved algorithms. However, there are two significant issues persist: (1) The mechanisms of welding defect prediction remain unclear. (2) Defect detection algorithms lack robustness across various welding scenarios. It makes the practical application of defect detection algorithms remain challenges.

In this paper, the molten pool sensing system has been developed, obtaining the clear molten pool images. The welding defects prediction model for GMAW process is established based on CNN. Furthermore, the mechanisms of welding defects in various welding scenarios are comprehensively analyzed based on welder experience. It contributes to enhancing the robustness of defect detection algorithms. The novel method for improving the model's robustness in defect detection is proposed. By highlighting welding features in input images based on welder experience and expanding the training dataset during various welding scenarios, the robustness of the CNN algorithm is significantly improved. The welding experiments in various welding scenarios were performed to validate the algorithm's robustness. This contributes to improving the practical application of GMAW defect detection. The welding defects detection during GMAW process is achieved accurately.

## Setup and experiment

### Welding robot controlling system

**Figure 1:**
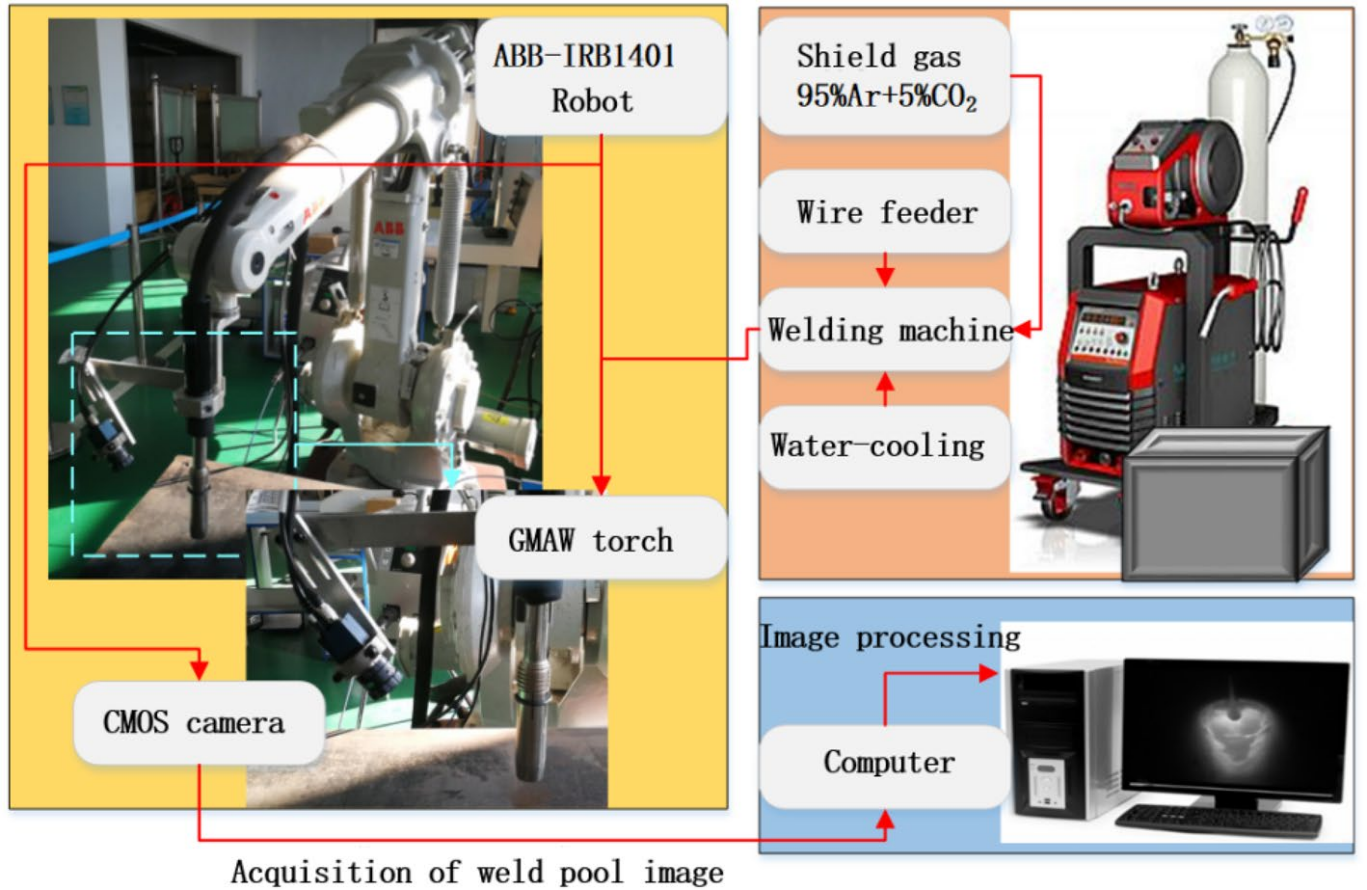
Schematic of GMAW experimental platform.

The GMAW experimental platform is shown as Fig. 1. It consists of the ABB robot, the control cabinet, the MAG welding machine and the wire feeder. The welding machine selected is MEGMEET welding machine and the model is ArtsenII PM500F. Other peripheral auxiliary equipment includes gas cylinder, pressure gauge, water-cooling welding gun. The welding machine and the robot control cabinet is connected with the relay by using IO serial port. The arc starting and arc stopping commands of the welding machine are controlled by using programming instructions.

### Vision sensing system

**Figure 2:**
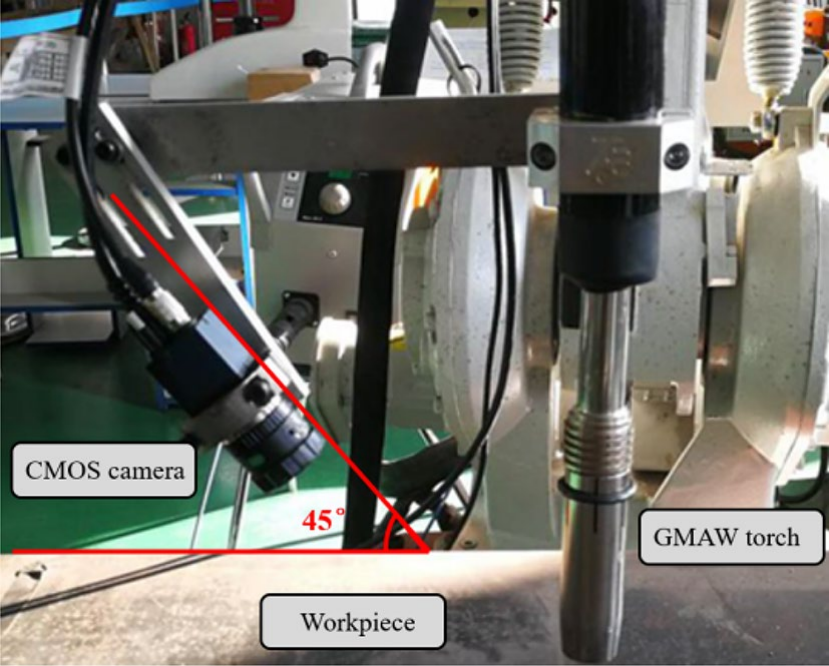
Relative position of camera welding torch.

The molten pool vision sensing system composes of the camera and the computer. The MER-231-41GM CMOS camera is adopted to monitor the molten pool. The filter is a narrow-band red light filter with a band range of 650 nm. The camera layout is shown in Fig. 2. The lens of CMOS is positioned at the distance of 150 mm from the welding gun and is settled at 45°horizontal angle to the workpiece. The camera and welding torch are fixed on the robot. The obtained molten pool image is processed by the developed image processing algorithm. The visual sensing system achieves the clear acquisition of molten pool image in real time through the optical path design.

### Experimental designs and result

The welding experiments under various welding conditions were performed to obtain molten pool images from different welding scenarios. The material is 022Cr19Ni10 stainless steel. The steel with specification of 200 × 50 × 3 mm is employed for the welding experiment with the butt joint. The butt gap is 0, 0.5 and 1 mm. The welding current and shielding gas flow are set as variables because the CNN algorithm requires a large dataset of image data. The target images are obtained through different combinations of welding parameters.

The main welding defect is sag depression, resulting from excessive welding heat input that increases the amount of base metal melting. The downward force gradually exceeds the surface tension of the molten pool. When the molten metal cannot be supported by the molten pool, it drips down under the influence of gravity, resulting in the penetration defect. The liquid metal flows out of the weld pool during penetration,, forming large dark welding crater within the weld pool. The backside of the weld seam has a through-hole. The front side of the weld seam exhibits noticeable concave feature due to the outflow of liquid metal. The general welding parameters of designed experiments are presented in Table 1.

**Table 1:** Design of welding experiment parameters.

Group	Gap	Electricity (A)	Shield gas flux (L/min)	Weld speed (mm/min)	Weld condition
1	0	90	2.5	150	No sag depression, pore, slag
2		90	20	150	No sag depression
3		95	2.5	150	No sag depression, pore, slag
4		95	20	150	No sag depression
5		100	2.5	150	No sag depression, pore, slag
6		100	20	150	No sag depression
7		110	2.5	190	No sag depression, pore, slag
8	0.5	110	20	190	Normal penetration
9		120	2.5	190	No sag depression, pore, slag
10		120	20	190	Partial sag depression
11		130	2.5	190	Partial sag depression, pore, slag
12		130	20	190	Sag depression
13		140	2.5	190	Sag depression, no pore, slag
14		140	20	190	Burn through
15	1	150	2.5	250	No sag depression, pore, slag
16		150	20	250	Burn through
17		160	2.5	250	Partial sag depression, pore, slag
18		160	20	250	Burn through
19		170	2.5	250	Burn through, pore, slag
20		170	20	250	Burn through
21		180	2.5	250	Burn through, pore, slag
22		180	20	250	Burn through

**Figure 3:**
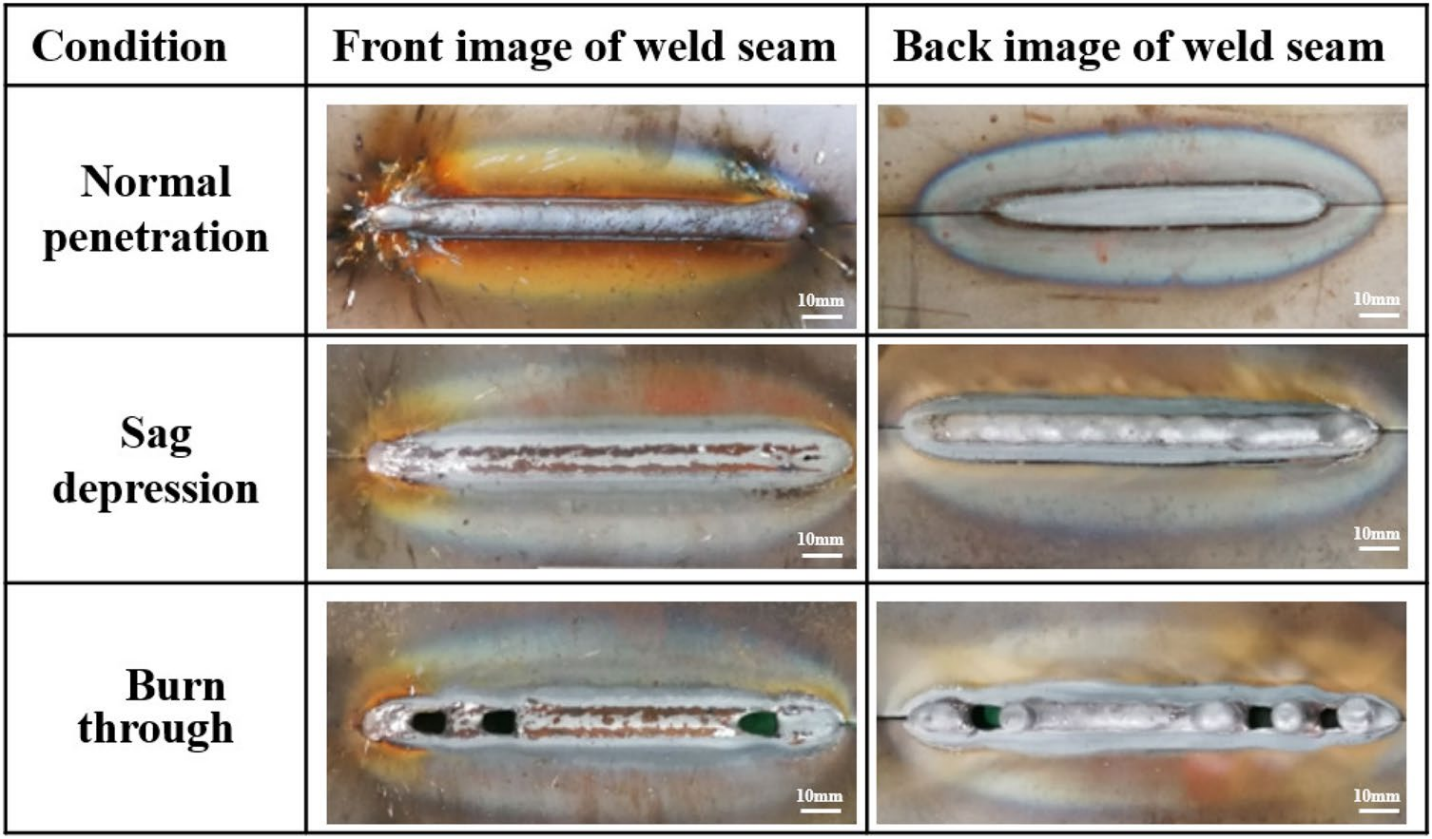
Weld morphology under different penetration conditions.

In the welding experiments, the welding current has great influence on the penetration state. The different penetration states are shown in Fig. 3.

The shielding gas flow rate affects the shielding effectiveness of the welding process. The shielding gas contributes to preventing the appearance of surface pores and slag defects. When the gas flow rate is more than 20L/min, the welding process achieves effective gas shielding. When the gas flow rate is less than 2.5 l/min, there are pores on the molten pool’s surface.

The molten pool image database is established to improve the accuracy and adaptability of the model. The welding process is recorded using the welding camera. The video frames are extracted as molten pool images to construct the dataset. The images at the beginning and the end of the welding process are excluded. The molten pool in the images are obscured due to the arc re-ignition caused by short-circuiting when the molten droplet is transferred. The arc interference images dataset is established to enhance the molten pool effects in the images by training the convolution model to classify the arc interference. The number of images in different classification databases is shown in Table 2. The training data is obtained through the image processing. The truth labels for molten pool images are assigned through welding experiments conducted at various weld depths. The welding images database is constructed for CNN algorithm. This welding images database serves as a universal resource for the development of welding penetration control models.

**Table 2:** Image acquisition.

Image library	Classification	Number of experiments	Number of images
Penetration	Full penetration	15	1650
	Sag depression	30	1850
	Burn through	15	1400
Pores	With pores	25	1350
	No pore	15	1050
Arc interference	Strong interference	100	10,000
	Weak interference	10	5300
Slags	With slags	15	2250
	Little slag	25	1950

## Image processing and database establishment of molten pool

### Image processing of molten pool

**Figure 4:**
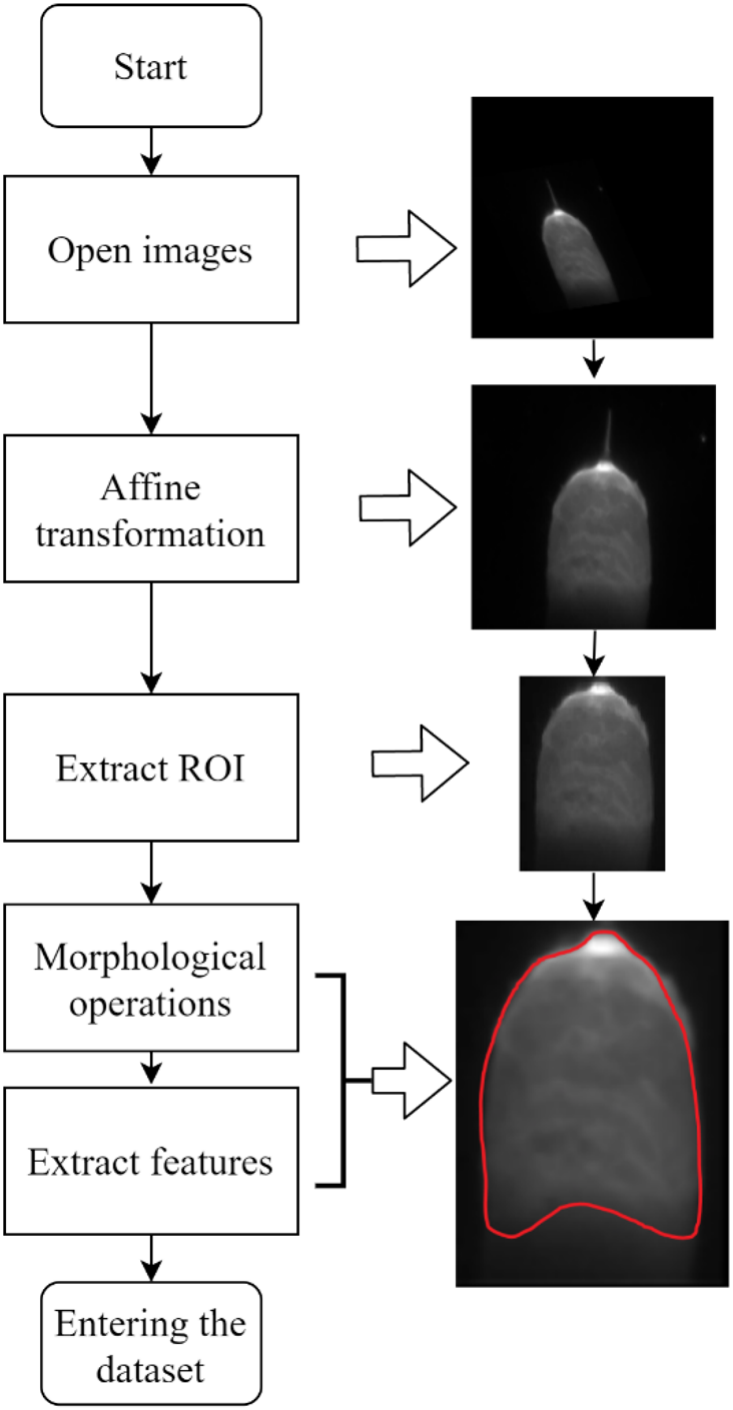
The steps of image processing.

The image processing workflow is illustrated in the Fig. 4.

**Figure 5:**
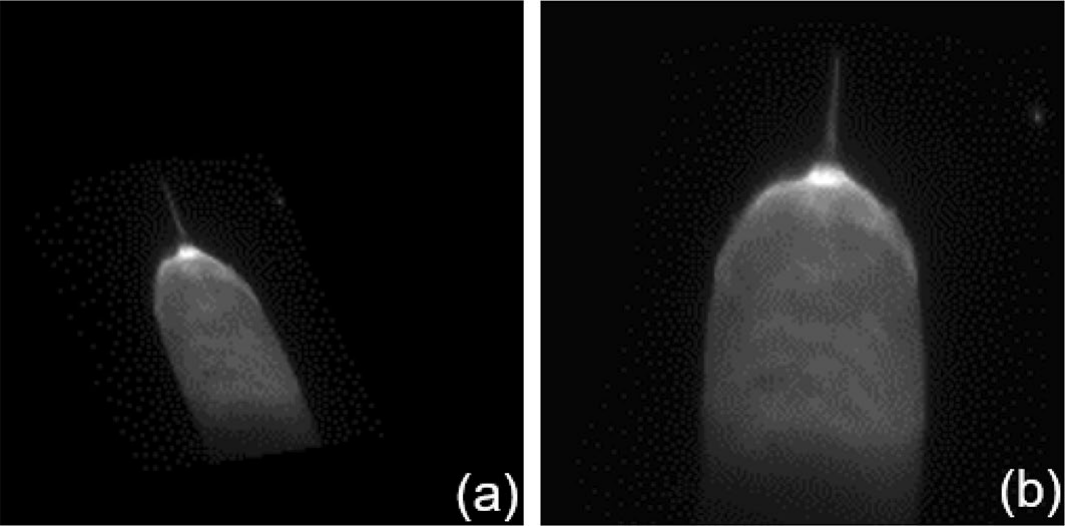
The comparison images of before and after calibration. (**a**) The image before the affine transformation. (**b**) The image after the affine transformation.

**Figure 6:**
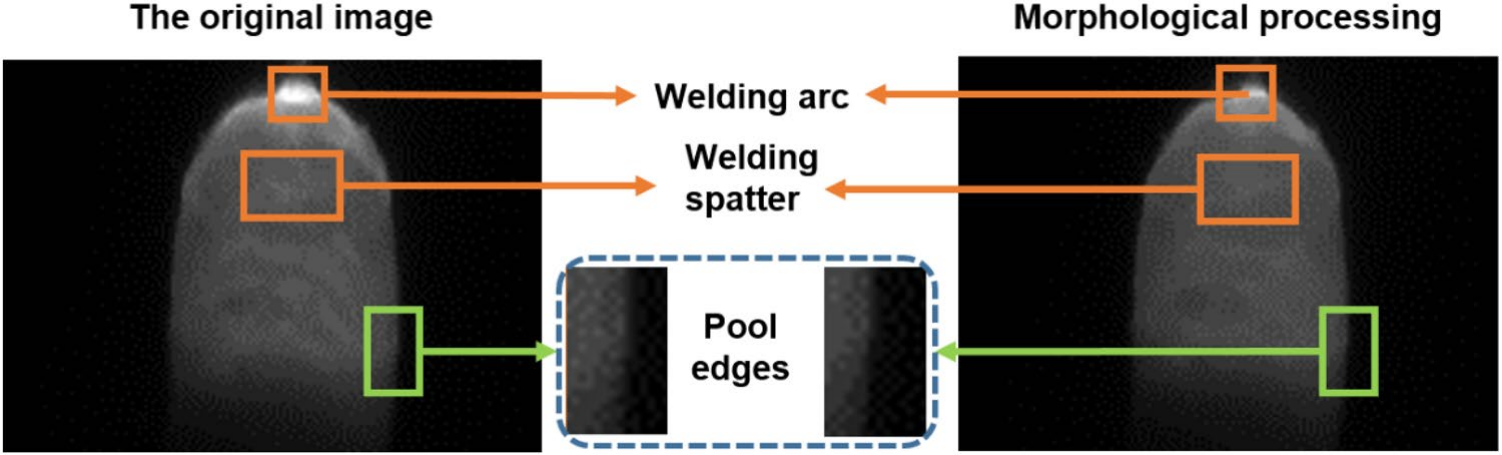
The comparison before and after morphological processing.

The region of interest(ROI) is defined within the original images to eliminate interference from the welding torch. There is still much interference information in the molten pool image such as welding spatter and arc interference, which are shown in Fig. 6. The distortion is reduced by transforming the image to a vertical angle through the affine transformations. The camera acquisition angle is fixed at 49.56 degrees, so the affine transformation matrix *K* is calculated as a fixed value. The comparison images of before and after affine transformation are shown in Fig. 5. The vertical-angle molten pool images are obtained after the affine transformation.

$$K = \left( {\begin{array}{*{20}c} {1.85717214 \, } & { - 0.81454918} & {43.00819593} \\ {0.26065572} & {1.62909835} & { - 86.01639186} \\ \end{array} } \right)$$

The morphological and Gaussian filter operation are employed on the ROI to get the clear image of molten pool. The erosion operations are utilized to diminish the bright areas in the images, reducing the impact of arc light. Then the dilation operations are used to eliminate the small black holes generated by spatter occlusions. The elliptical kernel is utilized as the mark shape in morphological opening operations for better preservation of edge details because the molten pool's edge resembles an ellipse. The mark size is set to 7*7. The comparison before and after morphological processing is shown in Fig. 6. The brightness of the arc and the welding spatter has been reduced. The low-gray oxide film area near the molten pool's edge is diminished. The edge details are well preserved. The molten pool images are included in the dataset after image processing.

### Image feature analysis of molten pool

**Figure 7:**
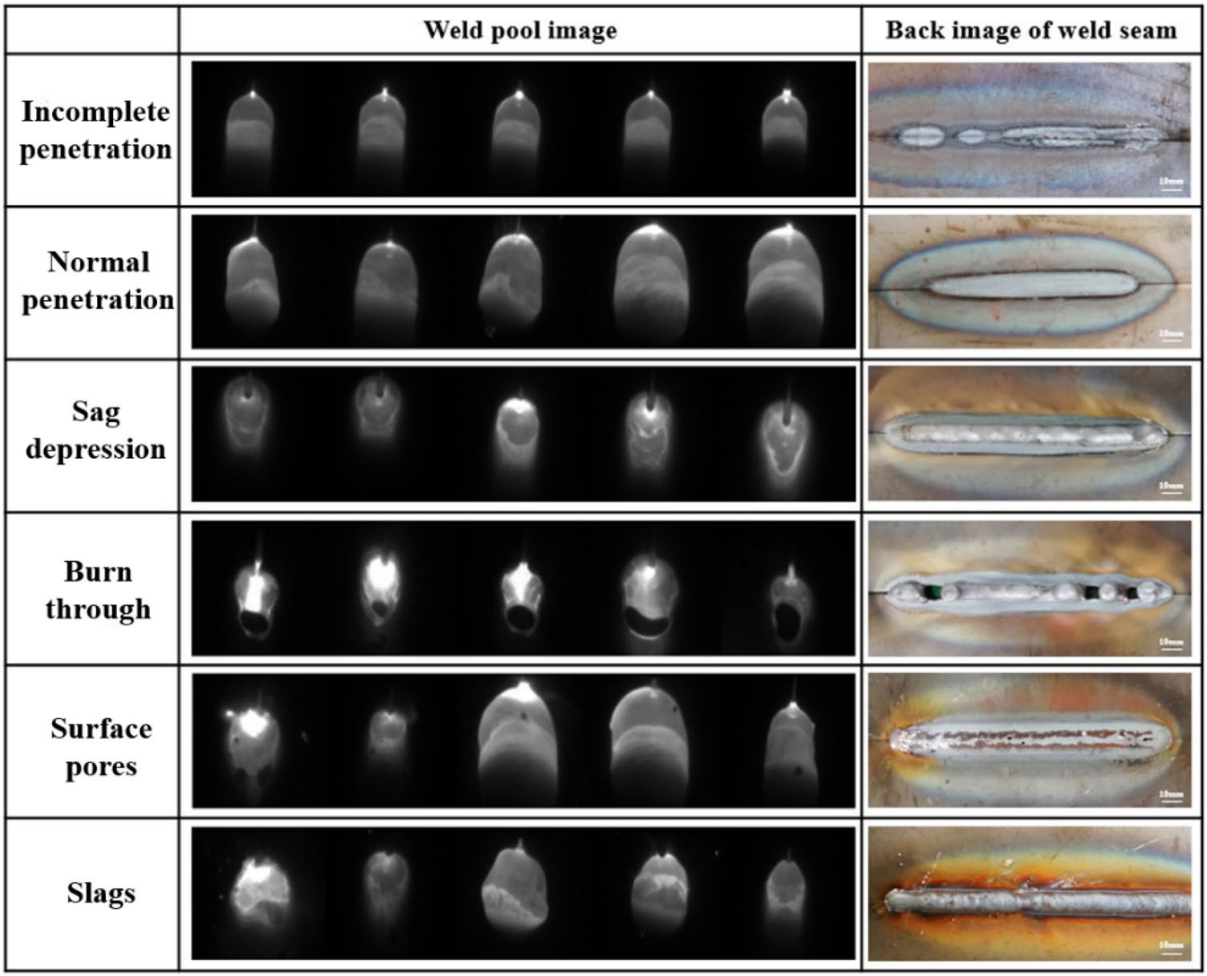
The image of molten pool and back of weld seam under different welding conditions.

The relationship between the characteristics of molten pool and defeats is analyzed based on image characteristics and welder experience. The images of molten pool and back of weld seam under different welding conditions are shown in Fig. 7.

The images of incomplete penetration and full penetration do not show significant feature distinctions. However, when the incomplete penetration occurs, the molten pool size is smaller due to the less heat input. The incomplete and full penetration are distinguished by the molten size. In the case of sag depression, the metal flow is obvious in the molten pool. The circular contour area appears in the tail of pool. When the burn through occurs, a large area of black weld craters appears in the weld pool. The weld craters are considered an important indicator of penetration state based on welder experience. The molten pool fails to maintain a steady flow due to high heat input, resulting in irregular variations in the welding crater size. In the case of surface pores, the gas bubbles reduce the reflection of arc light. There are black cavities between the liquid metal and the solidified metal at the tail of the molten pool. In the case of slags, the molten pool shape is irregular. The slags appeared on the weld seam surface if the welding specification and the gas protection effect is not suitable. It is dispersed by floating bubbles, resulting in small pieces at the rear edge of the molten pool.

### Establishment of image database

The large amount data processing method of loading data directly into memory slows down the calculation speed. TFrecord is the standard format of Tensorflow for reading and labeling the data which utilizing the memory effectively. The processed data is used as the input of the model to realize the detection of different welding defects.

## Prediction model of welding defects based on CNN

### Convolutional neural network

**Figure 8:**
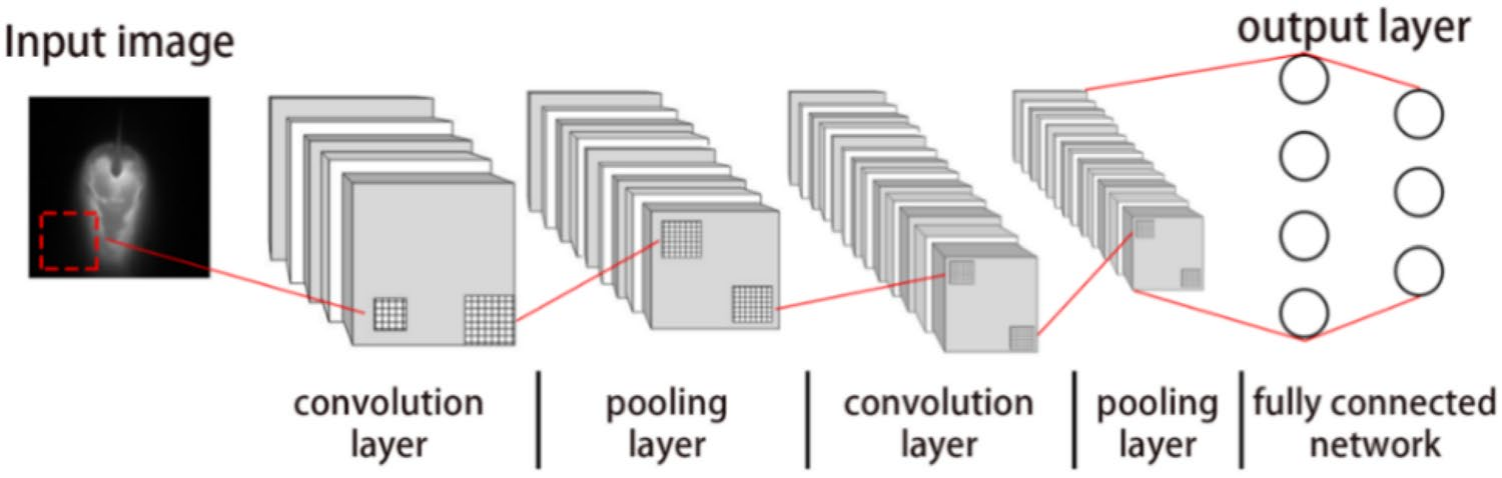
Schematic diagram of convolution neural network.

The image features of the molten pool with different welding defects are different. However, lots of features fail to be extracted through common image feature extraction algorithms such as the edge extraction. The classification of weld defects requires a combination of multiple features. CNN has an excellent performance in image classification. It effectively learn the corresponding features from a large number of samples without the complex feature extraction process. CNN designs the local connection conforming to the sparse response characteristics of biological neurons to avoid the redundancy of parameters caused by full connection between layers. The parameter size of the network and the dependence on training data are reduced. CNN mainly consists of three major layers: 1) convolution layer; 2) pooling layer; 3) full connection layer. The basic structure of CNN is shown in Fig. 8.

### Setting of CNN model training parameters

It is necessary to set the learning rate, batch size, convolution kernel size and iteration times for training convolutional neural network. Considering the computer performance, convergence speed, over fitting, a series of optional training parameters are obtained through experimental comparison and empirical parameters. The small dataset is conducted to perform the super parameter test for improving the experimental efficiency.

#### Learning rate

**Figure 9:**
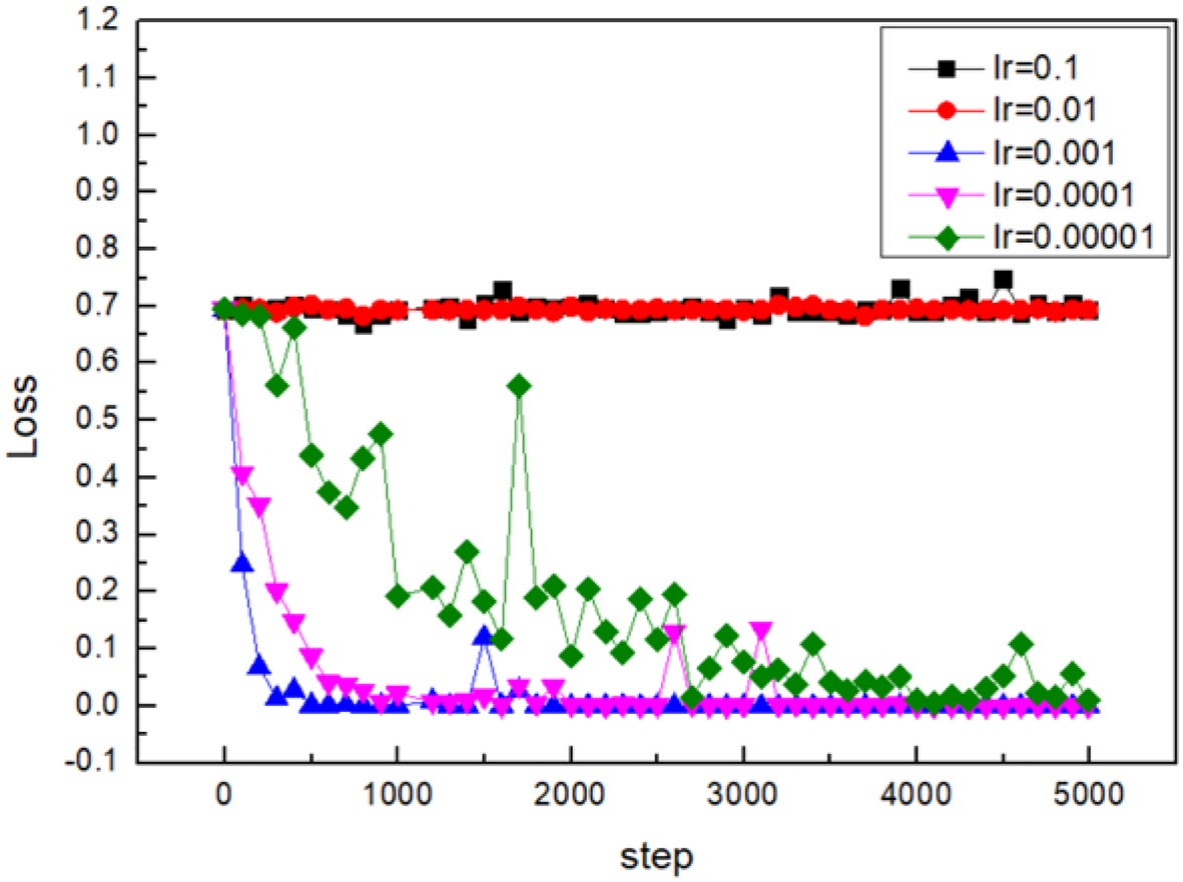
The influence of learning rate on the loss value.

The learning rate determines the extent to which weights move along the gradient direction within a batch. The training is easy to achieve stable convergence if the learning rate is low. However, the convergence takes a long time because the initial random weight is probably far away from the optimal value at the beginning. The training is hard to converge if the learning rate is high. The large weight variation reduces the optimization with the high learning rate. Therefore, it is of significance to get an appropriate learning rate. The CNN model is trained by enhanced stochastic gradient descent algorithm. For instance, the variations in loss of the surface pores prediction model with the training iterations are shown in Fig. 9.

#### Batch size

Batch size limits the number of pictures processed in each batch. The small batch size results in prolonged training times required for convergence. The large batch size results in insufficient computer video memory. The batch size is selected as 32 based on the data size and GPU training speed.

#### Convolution kernel size

**Figure 10:**
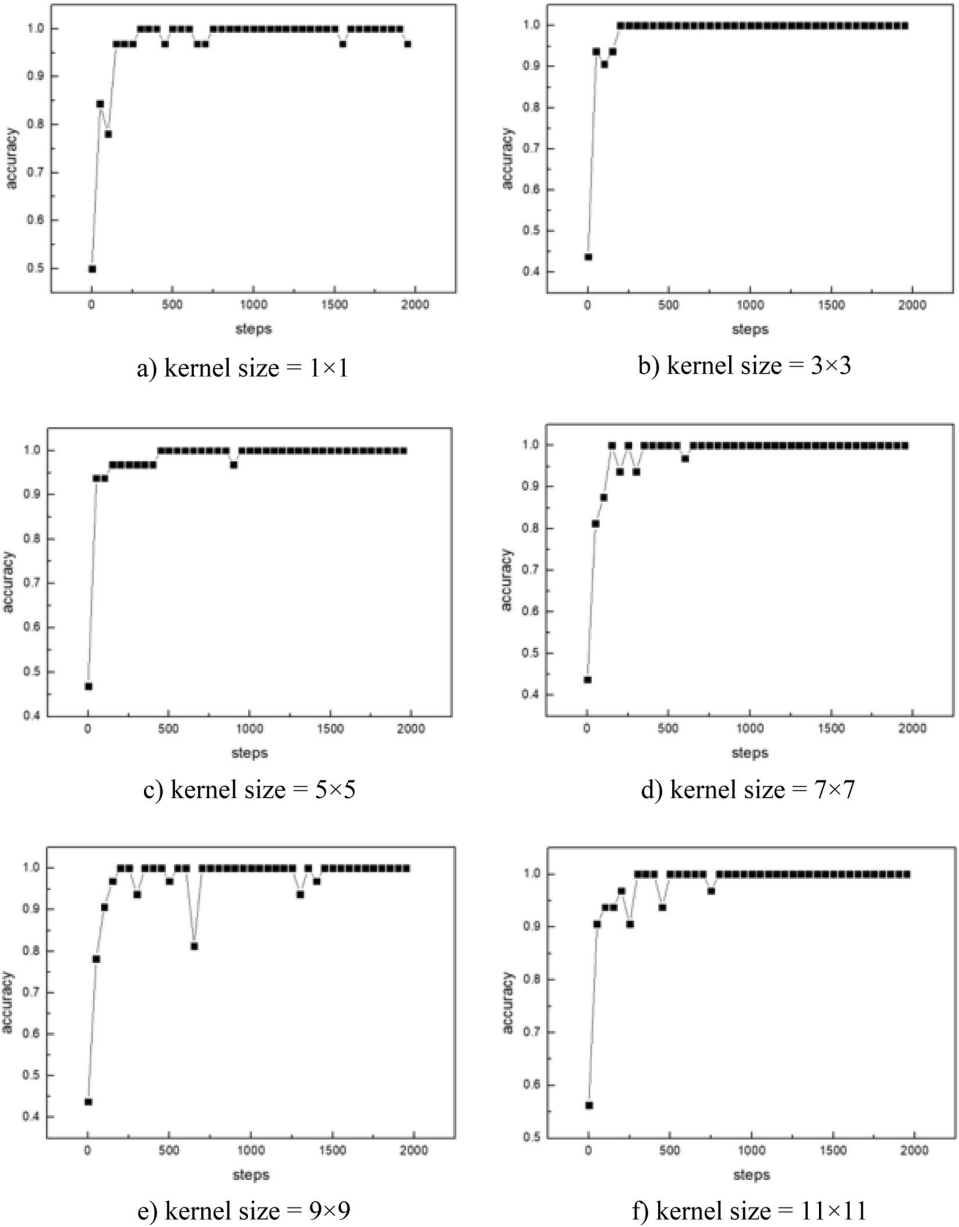
The accuracy of CNN model training.

Convolution kernel is the unit of convolution layer for obtaining image features. The first convolution kernel processes the input image and determines the size of receptive field during the first convolution. The optimal parameters are obtained through a comparison of different sizes of convolutional kernels. The final result is shown in Fig. 10.

The model has an excellent perform and converges quickly when the convolution kernel size is 3 × 3. Therefore, the first convolution kernel size is set as 3 × 3 in the CNN model.

#### Loss function and cost function

The disparity between predicted output *ŷ* and true value *y* is defined by the loss function. It quantifies the proximity of sample outputs to the true values during the training process. The cross entropy cost function (J) represents the average loss function value for the entire training dataset. It assesses the effectiveness of parameters *w* and *b* in fitting the training set. The training process involves iteratively optimizing and finding the corresponding *w* and *b* parameters to minimize the value of the cost function. The loss function and cost function are shown in formula 1.

1 $$\left\{ \begin{gathered} z = w^{T} x + b \hfill \\ y^{ \wedge } = a = g(z) \hfill \\ L(y^{ \wedge } ,y) = - \sum\limits_{i = = 1}^{K} {y_{i} \log ({y^{ \wedge }}_i )} \hfill \\ J(w,b) = \frac{1}{m}\sum\limits_{i = 1}^{m} {L(y^{ \wedge } (i),y(i))} \hfill \\ \end{gathered} \right..$$

### CNN model training process

**Figure 11:**
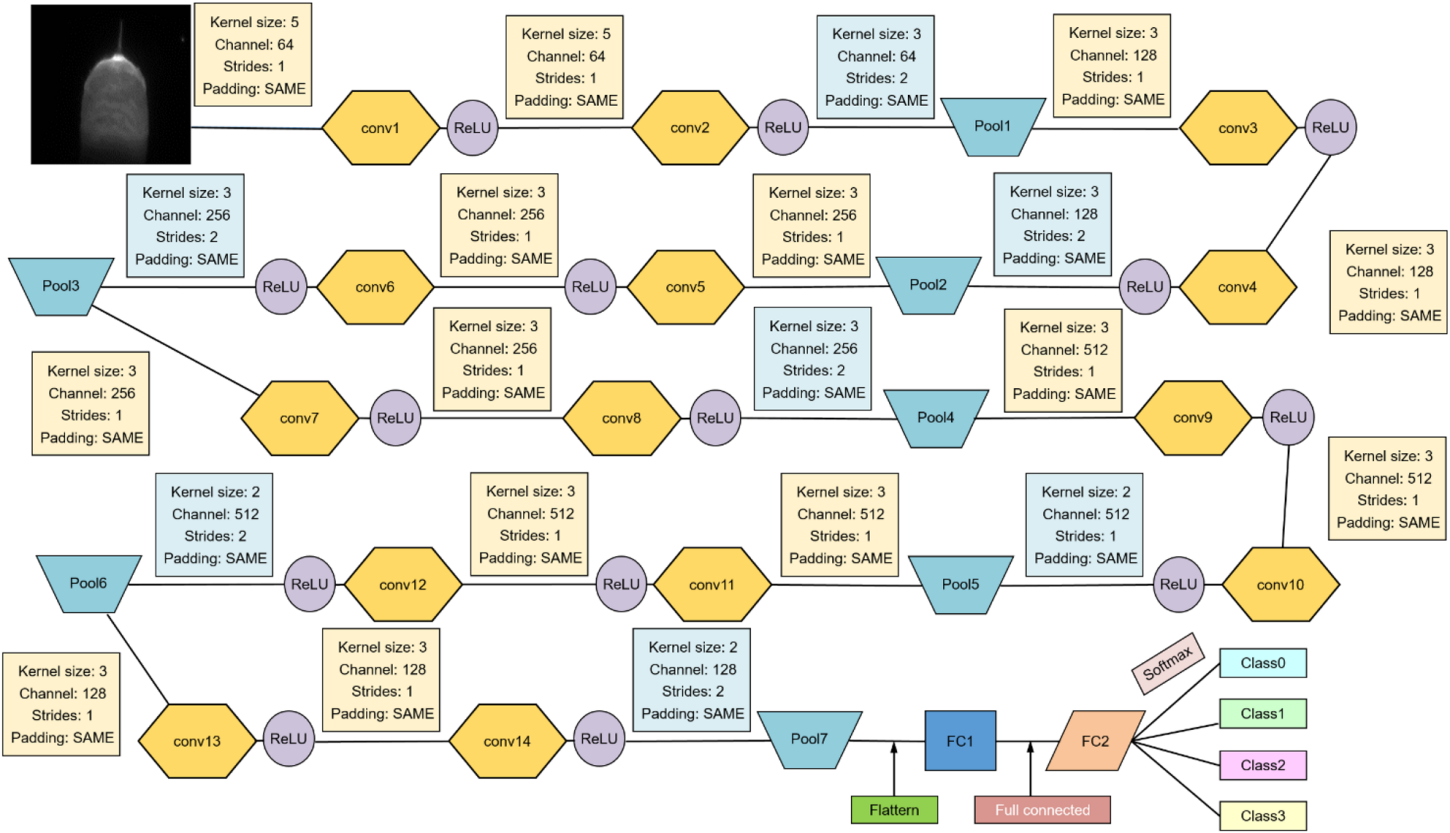
The CNN model architecture.

The CNN model architecture is shown in Fig. 11. The model comprises multiple sets of convolutional layers, pooling layers, the fully connected layer and output layer. The preprocessed molten pool image is input. The features are extracted through multiple layers of convolution and pooling. The fully connected layer and softmax layer are employed for classification.

The global training parameters of CNN network are shown in Table 3.

**Table 3:** Global training parameters of CNN network.

Parameter	Value
Image size	224 × 224
Batch size	32
Capacity	200
Max step	5000
Learning rate	0.001
Weight variable	0.005

**Figure 12:**
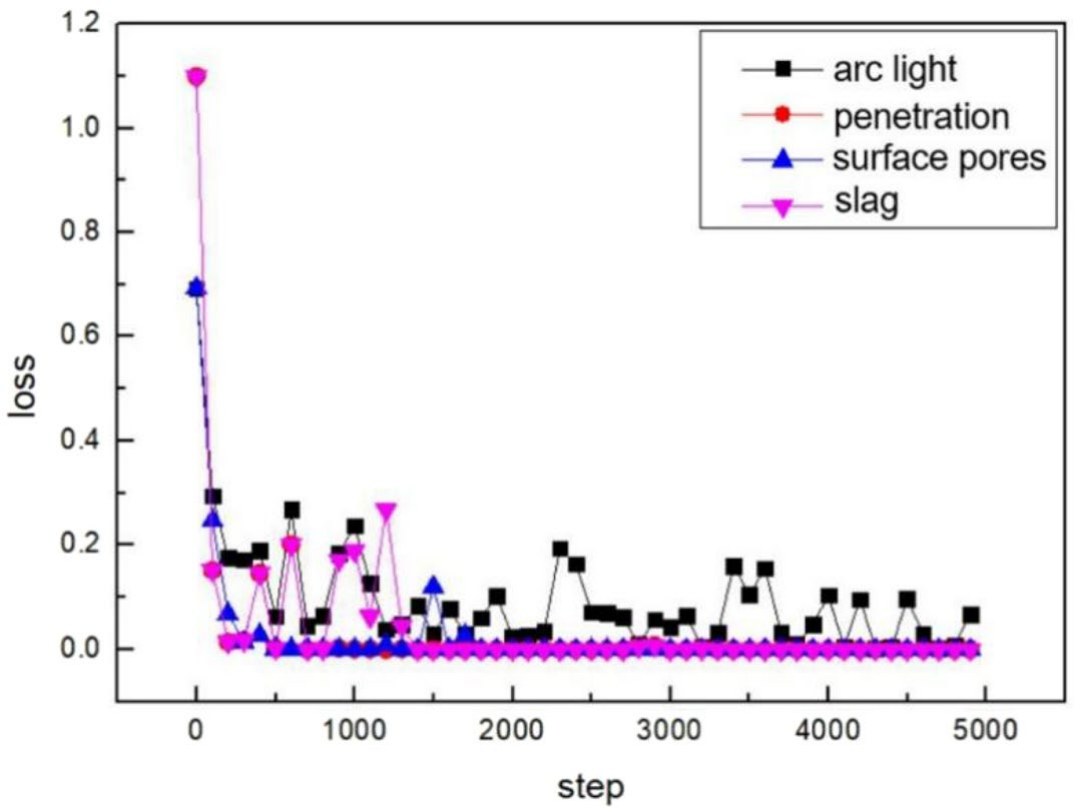
The change of the loss value.

**Figure 13:**
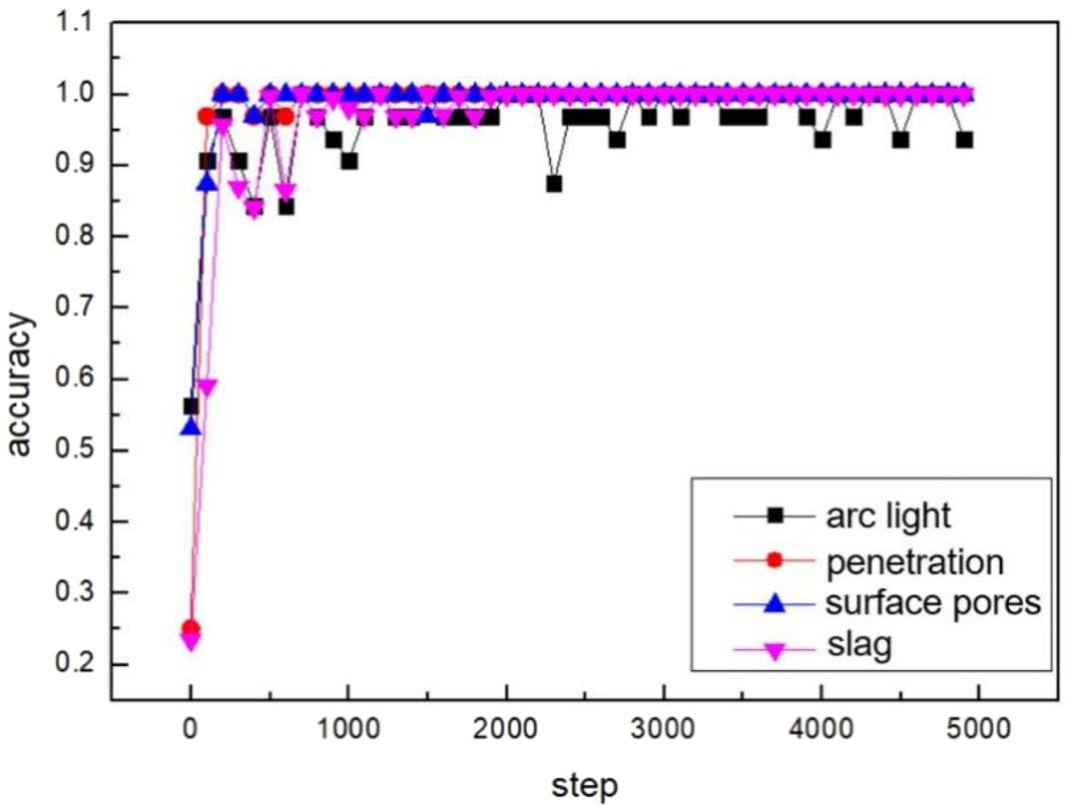
The change of the accuracy rate.

The four groups of labels are set, which consists of the arc interference intensity, welding penetration, surface pores and slag. The change of the loss value and accuracy rate in the training process with the increase of steps is shown in Figs. 12 and 13.

The loss value and accuracy rate of the penetration set, slag set, and surface porosity set converge rapidly and stably after 700 steps. The loss value is maintained at 0. The accuracy rate is maintained above 95%. However, the arc light set exhibits fluctuations in loss values between 0 and 0.2 even after convergence. Due to the overlapping area of arc interference, some images are hard to classify. The arc light dataset exhibits sparse gradients due to small differences between some images. Additionally, the objective function is non-stationary. The arc light model accelerates the convergence process by adopting an enhanced algorithm based on stochastic gradient descent (SGD). SGD approximates the average loss by randomly selecting a single training sample. It updates the weight values using only that single training data, significantly improving training speed. However, the randomness impacts the convergence performance.

The Adaptive Moment Estimation (Adam) algorithm is an improved algorithm used for optimizing stochastic gradient descent. It combines the concepts of momentum in gradient descent with adaptive learning rates to expedite convergence and better accommodate variations in gradient across different features. The Adam utilizes first and second moment information to dynamically adjust the learning rate for each parameter. The parameter *θ* update process is shown as in formula (2). Therefore, it exhibits an excellent performance in calculating sparse gradients and non-stationary objectives compared to other enhanced algorithms.

2 $$\Delta \theta_{t} = - \frac{{\frac{{\mu *m_{t - 1} + (1 - \mu )*g_{t} }}{{1 - \mu^{t} }}}}{{\sqrt {\frac{{v*\eta_{t - 1} + (1 - v)*g^{2}_{t} }}{{1 - v^{t} }} + \varepsilon } }}*\eta$$

**Figure 14:**
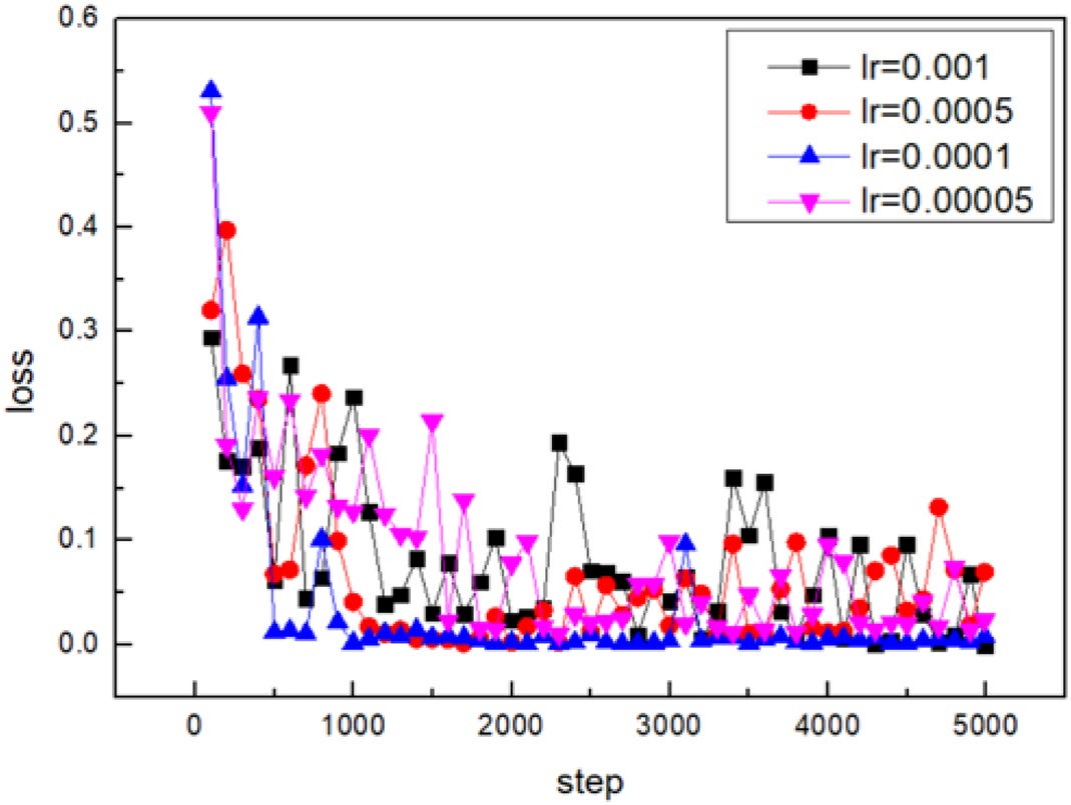
Fine tuning of learning rate for the arc light set.

The Adam algorithm is also used to avoid local optima points. It’s almost impossible for the entire model to get stuck in a local optimum for the deep neural networks with numerous parameters. For the common points during the training process where gradients are close to zero, The Adam algorithm allows for rapid movement away from these points due to its parameter update methods. The Adam algorithm demonstrates fast and stable convergence due to the presence of a large number of sparse gradients in the arc light model. The training process with representative learning rates is shown in Fig. 14.

**Figure 15:**
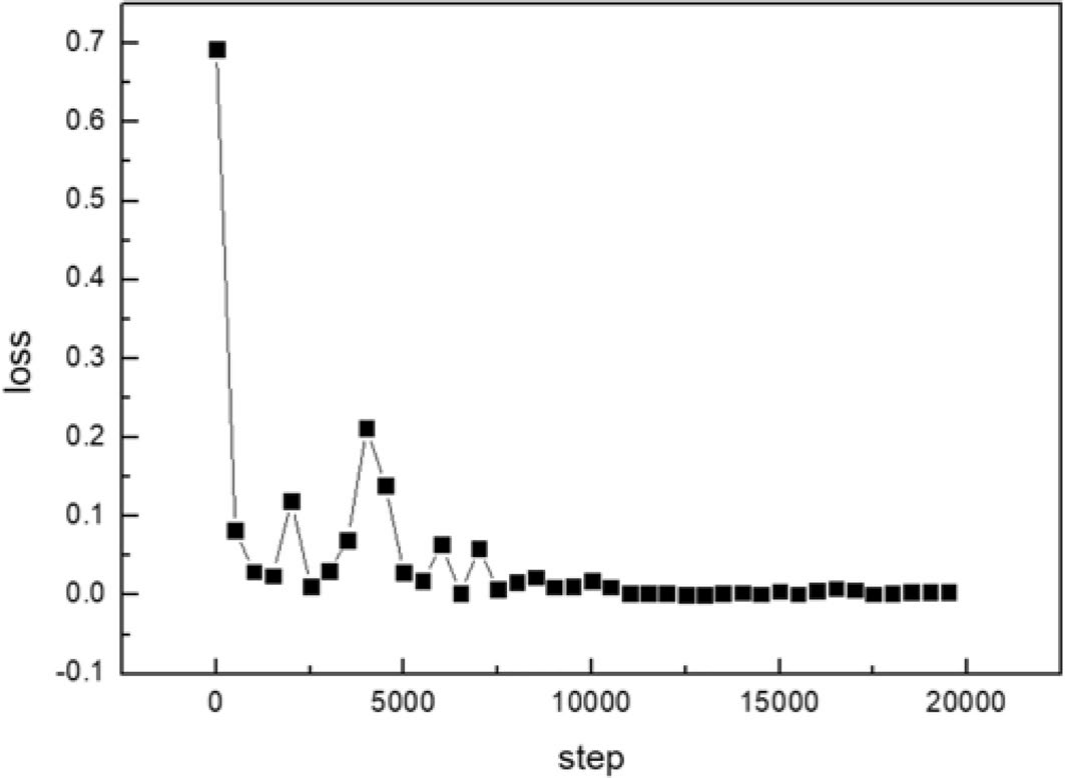
Fine tuning of iterations for the arc light set.

The model achieves stable convergence when the LR value is set to 0.0001 and the iteration number is set to 12,500, which are shown in Fig. 15.

### Performance analysis of model prediction

The CNN model architecture is shown in Fig. 11.The model consists of 14 convolutional layers, 7 pooling layers, and 3 fully connected layers. The architecture of the fully connected layers is divided into 7 layer groups, each comprising two convolutional layers followed by a pooling layer. The ReLU function is used as the activation function throughout the model. The input weld pool image has dimensions of 55 × 100 × 1. In the first layer group, 64 convolutional filters of size 5 × 5 are applied to the input image with a stride of 1. The first pooling layer uses a window size of 3 for max-pooling with a stride of 2. In the second layer group, 128 convolutional filters of size 3 × 3 are used with a stride of 1. A pooling layer with a window size of 3 and a stride of 2 is followed. The third layer group employs 256 convolutional filters of size 3 × 3 with a stride of 1. The third pooling layer uses a window size of 3 with a stride of 2. In the fourth layer group, there are 256 convolutional filters of size 3 × 3 with a stride of 1. A pooling layer with a window size of 3 and a stride of 2 is followed. The fifth layer group utilizes 512 convolutional filters of size 3 × 3 with a stride of 1. The fifth pooling layer uses a window size of 2 with a stride of 2. In the sixth layer group, there are 512 convolutional filters of size 3 × 3 with a stride of 1. A pooling layer with a window size of 2 and a stride of 2 is followed. The seventh layer group consists of 512 convolutional filters of size 3 × 3 with a stride of 1. The seventh pooling layer uses a window size of 2 with a stride of 2. Then the data is combined to a one-dimensional vector and forms the first fully connected layer. The second fully connected layer and the output layer are connected using the softmax function for classification. The final output is the four types of molten pool conditions. The classification is achieved through the unified CNN model, which takes the molten pool images as input and outputs the detected defects.

The CNN model predicts arc interference, weld penetration, surface pores and slag generation precisely. The images in the test set are predicted by the model. The prediction difficulty varies for different defects based on welding experience and molten pool images. The images of the normal penetration state is similar to the incomplete penetration. The images of the pores and slag defect have less distinct features. The proportion of these dataset in the total dataset is set higher to improve training and testing accuracy. The results are shown in Table 4.

**Table 4:** CNN Model test results.

Test set	Classification	Number of pictures (sheet)	Accuracy	Test speed (s/sheet)
Penetration	Full penetration	362	0.995945	0.060629
	Sag depression	224	0.989011	
	Burn through	133	0.993358	
Pores	With pores	305	0.971365	0.060836
	No pore	295	0.980151	
Arc light	Strong arc interference	1121	0.996725	0.060782
	Weak arc interference	560	0.971772	
Slags	With slags	595	0.985129	0.060645
	Little slag	300	0.970987	

The results demonstrate that demonstrate that the accuracy of the four prediction models are all over 97%. The model classifies the input molten pool images accurately. The arc interference, penetration status, surface pores and slags generation are assessed quickly. The model exhibits an excellent performance in the prediction of the weld defects.

Considering the welding speed of 2.5 mm/s and the visual inspection requirement for welding defects lengths to be less than 1 mm. The real-time feedback during the welding process is required to be completed within 0.4 s, which is the time required to weld a length of 1 mm at the welding speed.

**Figure 16:**
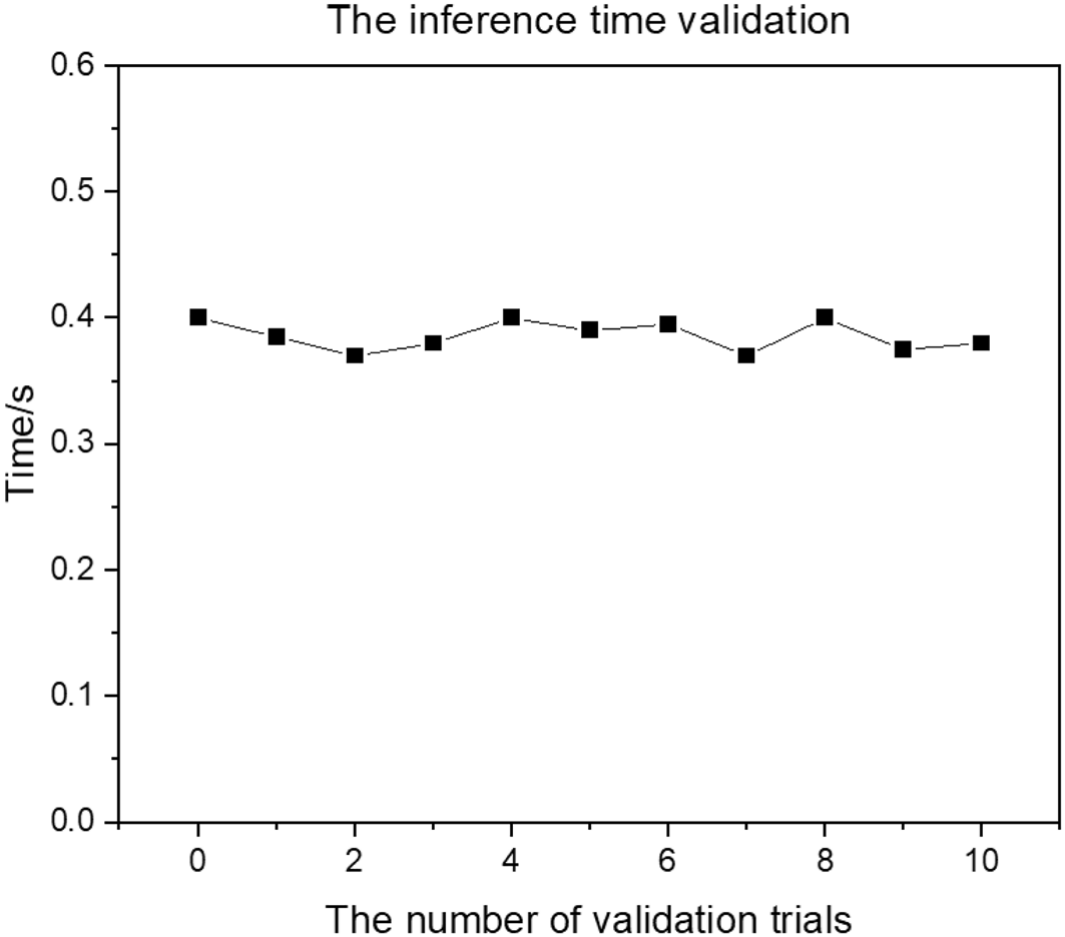
The CNN model's inference time validation.

The time validation experiment is conducted to verify the inference time of the CNN model. The experiment involves performing 11 random inference process, as shown in the Fig. 16. The average inference time is 0.38 s, which meets the requirement for real-time feedback during the welding process.

### Feature visualization

The CNN inputs the molten pool images and extracts local features automatically. Feature mapping is the result of convolution operation using a convolution kernel to the previous image layer. The features which play a key role in image classification are analyzed by using feature mapping.

#### Feature visualization in the penetration state

**Figure 17:**
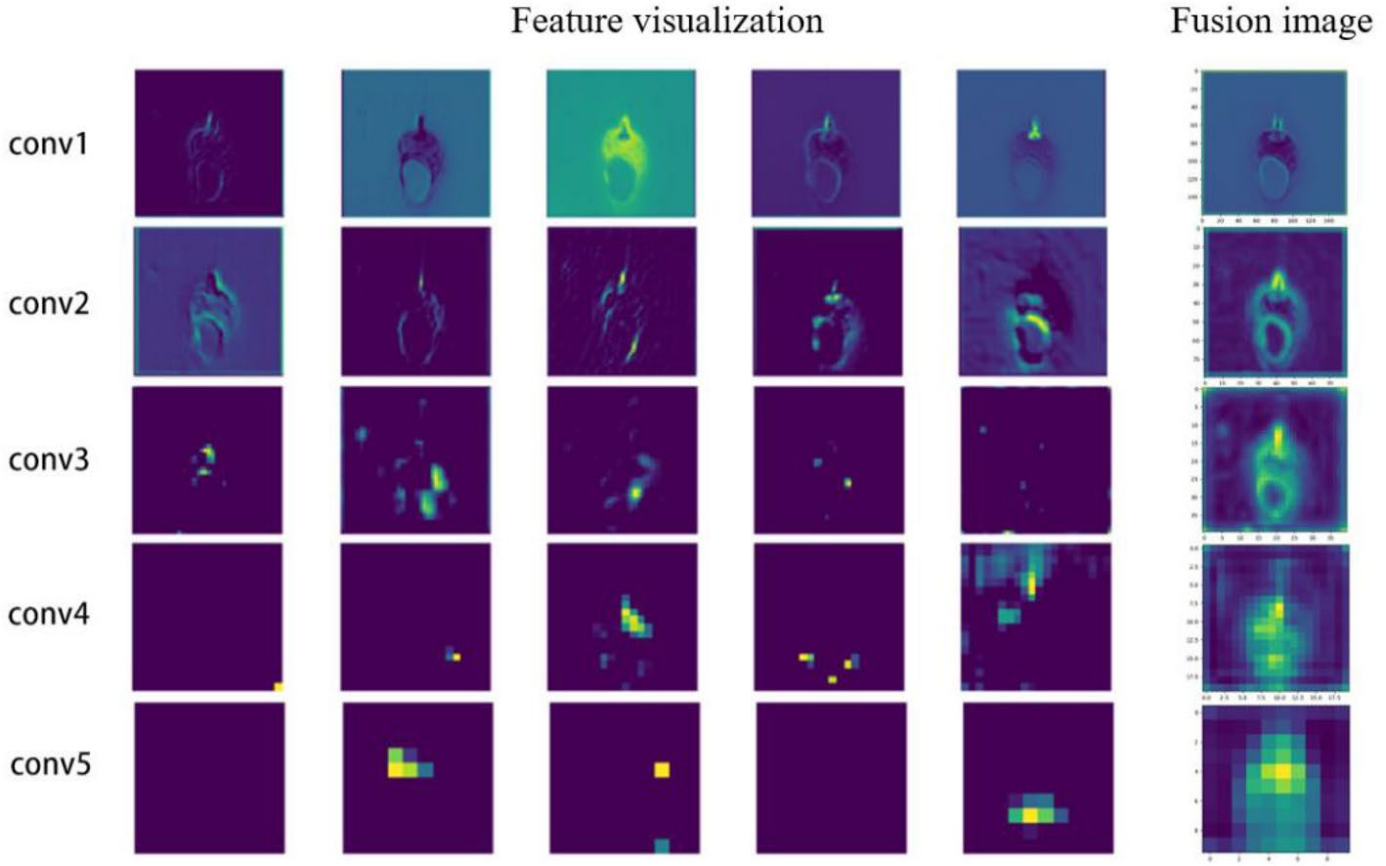
Feature visualization in the burn-through state.

The molten pool images in different penetration states processed by convolution kernel are visualized. The feature visualization of the molten pool image and the fusion image in the burn-through state are shown in Fig. 17. The first layer performs the function of edge detection to extract the contour of molten pool. The second layer extracts the features from the molten pool's tail area, including its shape and the weld seam behind it. The third and fourth layer focus on local information, such as the top, tail, and bright reflections of the arc light in the image. The last layer abstracts and encodes the extracted features to serve as the basis for model classification and recognition.

**Figure 18:**
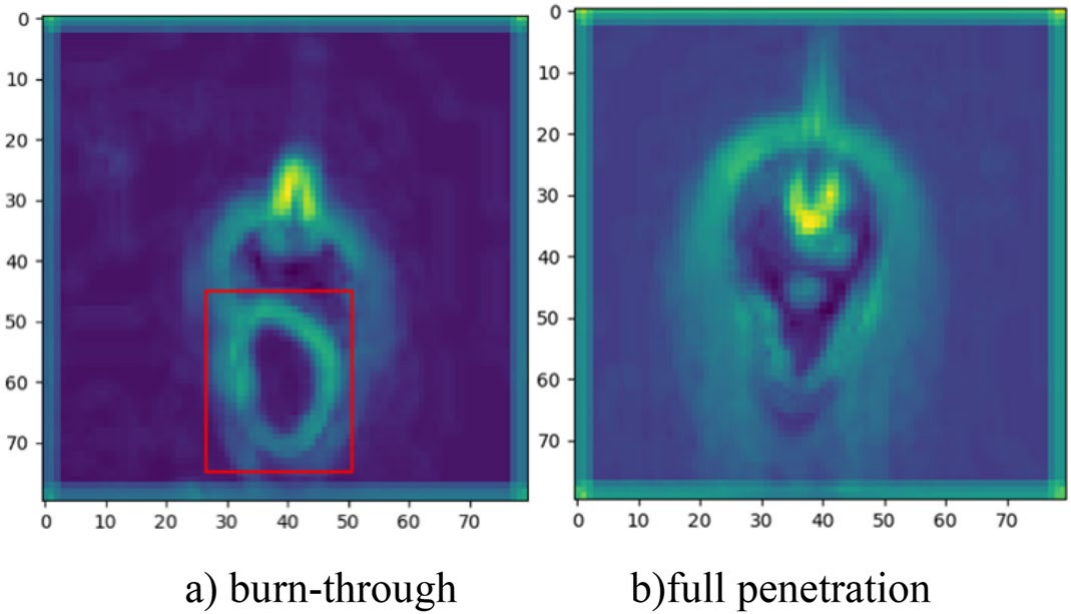
The comparison of burn-through and normal penetration feature visualization.

**Figure 19:**
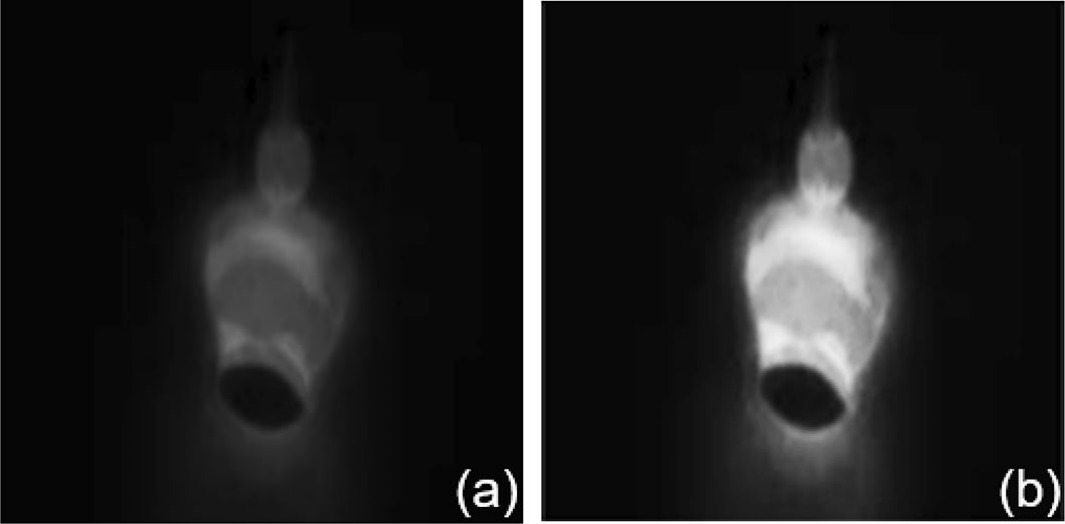
The comparison of fusion hole highlighting before and after. (**a**) The image before fusion hole highlighting. (**b**) The image after fusion hole highlighting.

The image in the burn-through state exhibit significant differences compared to the image in the full penetration, which are shown in Fig. 18. The black fusion holes are extracted in the burn-through images which are considered a sign of burn-through based on welding experience. The CNN model places a higher emphasis on fusion holes by highlighting the fusion hole area when processing burn-through images, which are shown in Fig. 19. The model is guided to learn the characteristics of fusion holes based on welder's experience which is distinct from traditional CNN models. The results (as shown in Table 4) on the test sets from different welding conditions demonstrated the model's robustness, achieving an accuracy of 99.33%.

**Figure 20:**
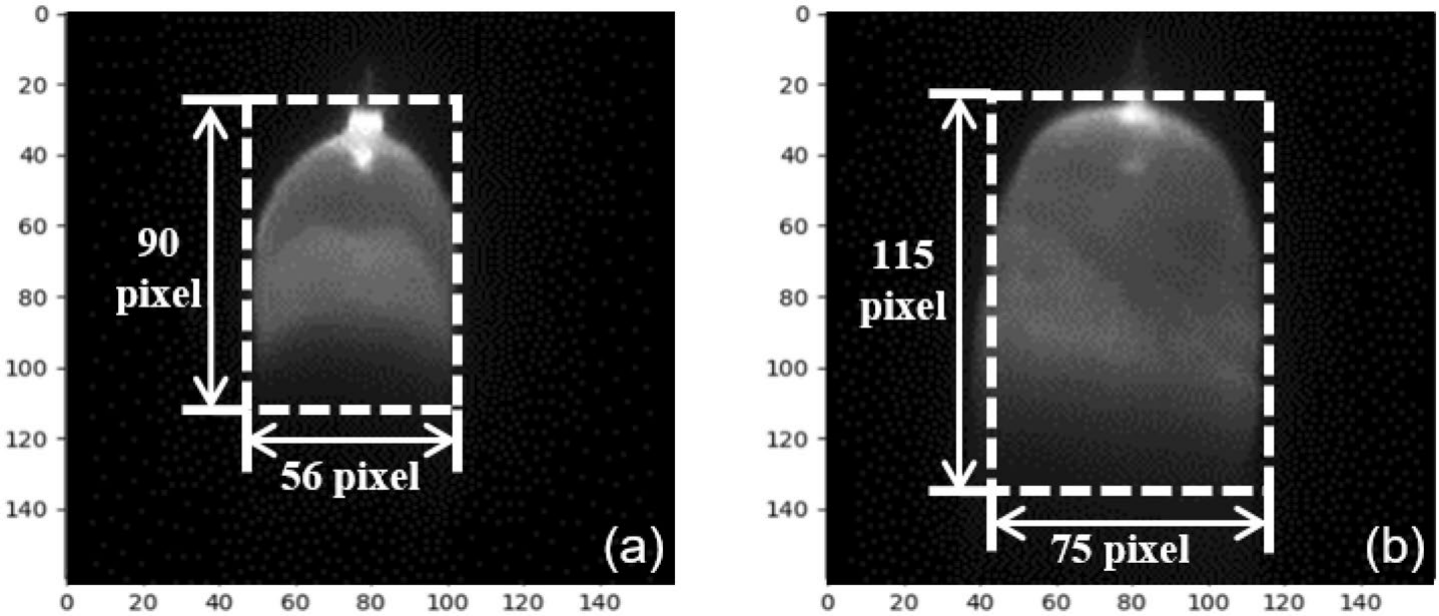
The comparison of incomplete penetration and full penetration. (**a**) The images of incomplete penetration. (**b**) The images of full penetration.

The images of incomplete penetration and normal penetration do not show significant feature distinctions. The molten pool size of the images is the important distinguishing factor due to the varying heat inputs, which is shown in Fig. 20. The molten pool area of incomplete penetration is much smaller than the full penetration due to insufficient heat input. The area of the molten pool in the incomplete penetration images is less than 5500 px^2^, while it is more than 8500 px^2^ in the full penetration images. The model predicts the state of penetration accurately based on the molten pool size.

#### Feature visualization in the surface pores state

**Figure 21:**
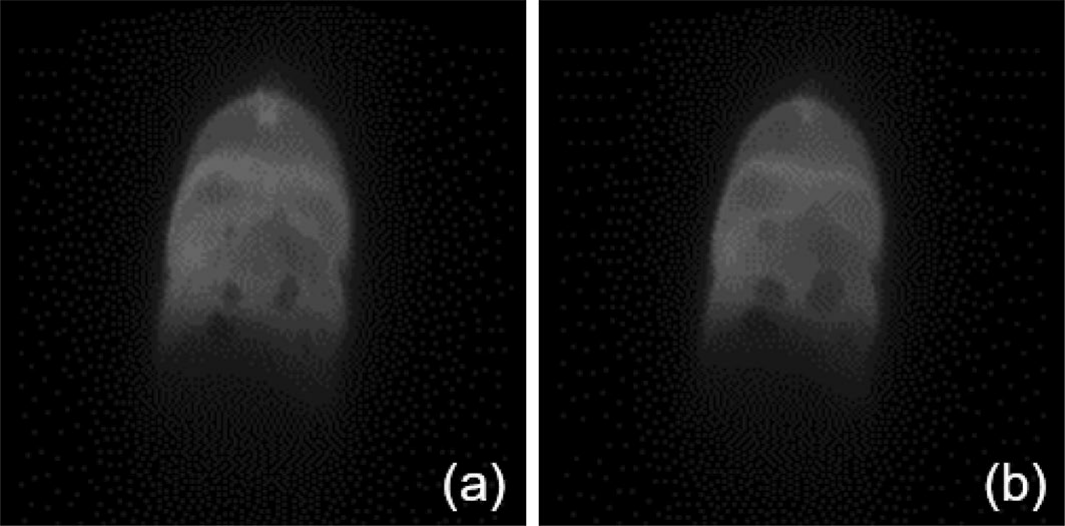
The comparison before and after dilation operation. (**a**) The image before dilation operation. (**b**) The image after dilation operation.

**Figure 22:**
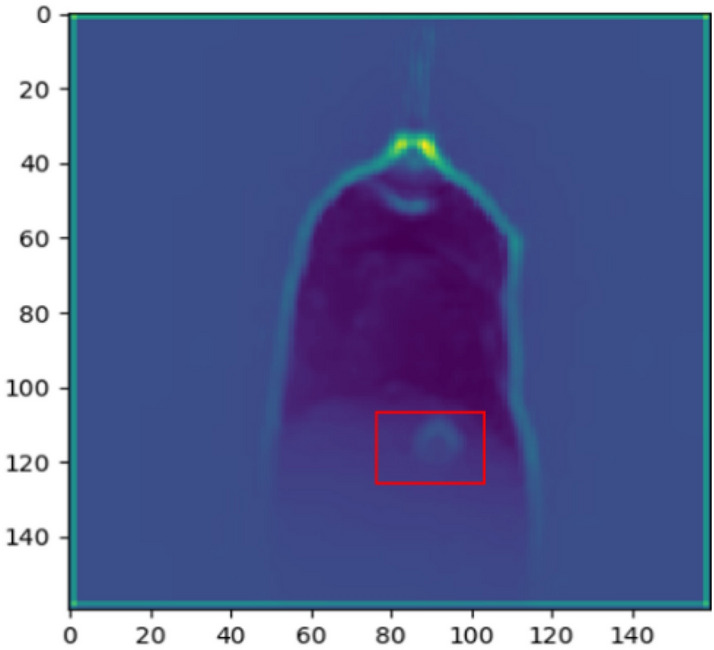
Feature visualization in the surface pores state.

The small surface pores is missed easily at the low image resolutions. The dilation operation is carried out to enlarge the pore feature for the feature extraction of convolution kernel. The elliptical kernel is utilized as the mark shape in the dilation operations for better preservation of edge details because the pore's edge resembles an ellipse. The mark size is set to 5*5. The comparison before and after dilation operation is shown in Fig. 21. The pores in the images are enlarged while the details of the molten pool are preserved. The model is guided to learn the small pore features in the pore defect images. The feature visualization image of the molten pool image with surface pores is shown in Fig. 22. The black hole appears in the partially solidified region at the tail of the molten pool. The convolution kernel has strong response in this region. The surface pores characteristics are identified successfully.

#### Feature visualization in the slag state

**Figure 23:**
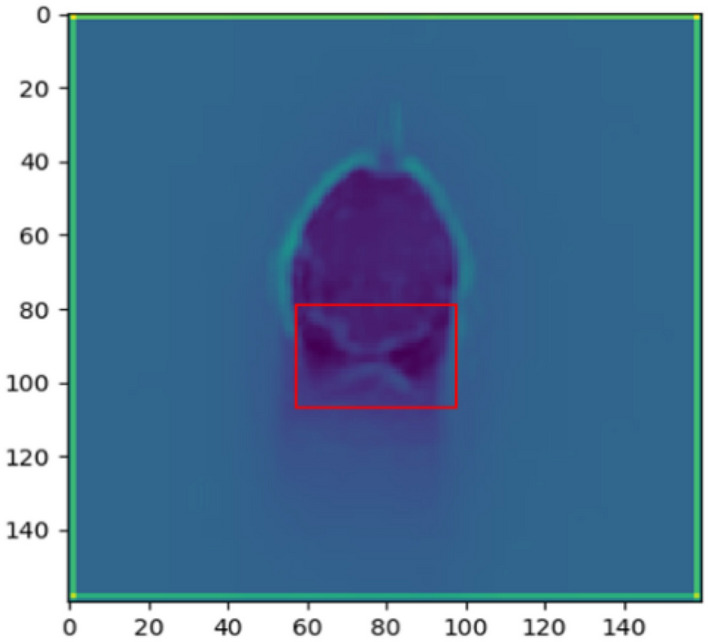
Feature visualization in the slag state.

The feature visualization of the slag state is shown in Fig. 23. There is an obvious boundary between the slag and the molten pool metal. The generated slag flows to both sides of the weld bead and accumulates. The convolution kernel has a strong characteristic response in the boundary region. The surface slag is detected accurately.

The prediction model distinguishes the classification features by accessing the edge of the molten pool, the welding feature region and the relative position between the regions. The detection process is similar to welders. The model achieves the accurate detection of various welding defects. The smoke and image interference fringes have little impact on the convolutional kernels, indicating that the model has a strong anti-interference capability. Moreover, the experimental validation of defect detection demonstrates that the model exhibits great robustness to various welding scenarios.

## Conclusion

In this paper, an intelligent model based on convolutional neural network is presented to detect defects in GMAW process in real time. The model predicts the penetration state, surface pores and slag accurately. The robustness of the defect detection model has been improved by enhancing the focus on welding features based on the welder experience. The model improves the accuracy performance during various welding scenarios. The following conclusions are summarized:

1. The visual sensing system of molten pool is built and the image processing algorithm is developed to obtain clear molten pool images. The arc interference images dataset is established to enhance the molten pool effects in the images by training the convolution model to classify the arc interference.
2. The parameters of the defect detection model are researched. The influence of different parameters on prediction accuracy is analyzed. The optimal network parameters are determined as follows: the first layer convolution kernel size is 3 × 3, there are three convolution layers, three pooling layers, and two full connection layers.
3. The defect detection model is developed based on the welding experience and CNN algorithm. The CNN model places a higher emphasis on fusion holes by highlighting the fusion hole and pores area when processing input images. The model is guided to learn the welding characteristics based on welder's experience which is distinct from traditional CNN models. The experiments for defect detection in different welding scenarios are conducted. The results demonstrate that the algorithm exhibits good robustness. The accuracy of the four defect prediction is over 97%. The average accuracy of judging various penetration states is more than 99%. The average inference time is 0.38 s, which meets the requirement for real-time feedback during the welding process. The real-time defect detection in GMAW has been achieved precisely.
4. The relationship between the image features of molten pool and penetration state, surface pores and slag is researched. The model accesses the state of penetration with the fusion hole in the middle zone of the molten pool. The state of penetration is predicted accurately based on the molten pool size. The black hole appears in the partially solidified region at the tail of the molten pool in the state of surface pores. The convolution kernel has strong response in this region. The model has a strong characteristic response in the boundary region between the molten pool metal and slags when the slags are generated. The prediction model distinguishes the classification features by accessing the edge of the molten pool, the welding feature region and the relative position between the regions. The detection process is similar to welders. The robust detection of various welding defects is achieved. It contributes to promoting the practical application of GMAW defect detection.

## Data Availability

The datasets generated and analyzed during the current study are not publicly available due the confidentiality of the data but are available from the corresponding author on reasonable request.
